# Genetic Link Determining the Maternal-Fetal Circulation of Vitamin D

**DOI:** 10.3389/fgene.2021.721488

**Published:** 2021-09-21

**Authors:** Aparna Sampathkumar, Karen M. Tan, Li Chen, Mary F. F. Chong, Fabian Yap, Keith M. Godfrey, Yap Seng Chong, Peter D. Gluckman, Adaikalavan Ramasamy, Neerja Karnani

**Affiliations:** ^1^Singapore Institute for Clinical Sciences (SICS), Agency for Science, Technology and Research (A*STAR), Singapore, Singapore; ^2^Saw Swee Hock School of Public Health (SSHPH), National University of Singapore (NUS), Singapore, Singapore; ^3^Department of Pediatric Endocrinology, KK Women’s and Children’s Hospital, Singapore, Singapore; ^4^Duke-NUS Medical School, Singapore, Singapore; ^5^Lee Kong Chian School of Medicine, Singapore, Singapore; ^6^MRC Lifecourse Epidemiology Unit, University of Southampton, Southampton, United Kingdom; ^7^NIHR Southampton Biomedical Research Centre, University of Southampton and University Hospital, Southampton, United Kingdom; ^8^Department of Obstetrics and Gynecology, Yong Loo Lin School of Medicine, National University of Singapore, Singapore, Singapore; ^9^Liggins Institute, University of Auckland, Auckland, New Zealand; ^10^Genome Institute of Singapore (GIS), Agency for Science, Technology and Research (A*STAR), Singapore, Singapore; ^11^Department of Biochemistry, National University of Singapore (NUS), Singapore, Singapore; ^12^Bioinformatics Institute (BII), Agency for Science, Technology and Research (A*STAR), Singapore, Singapore

**Keywords:** vitamin D, ethnicity, genome-wide association study, pregnancy, offspring, GUSTO

## Abstract

Vitamin D is an essential micronutrient whose demand is heightened during pregnancy to support the growth of the fetus. Furthermore, the fetus does not produce vitamin D and hence relies exclusively on the supply of maternal vitamin D through the placenta. Vitamin D inadequacy is linked with pregnancy complications and adverse infant outcomes. Hence, early predictive markers of vitamin D inadequacy such as genetic vulnerability are important to both mother and offspring. In this multi-ethnic Asian birth cohort study, we report the first genome-wide association analysis (GWAS) of maternal and fetal vitamin D in circulation. For this, 25-hydroxyvitamin D (25OHD) was measured in the antenatal blood of mothers during mid gestation (*n*=942), and the cord blood of their offspring at birth (*n*=812). Around ~7 million single nucleotide polymorphisms (SNPs) were regressed against 25OHD concentrations to identify genetic risk variants. About 41% of mothers had inadequate 25OHD (≤75nmol/L) during pregnancy. Antenatal 25OHD was associated with ethnicity [Malay (*Β*=−22.32nmol/L, *p*=2.3×10^−26^); Indian (*Β*=−21.85, *p*=3.1×10^−21^); reference Chinese], age (*Β*=0.47/year, *p*=0.0058), and supplement intake (*Β*=16.47, *p*=2.4×10^−13^). Cord blood 25OHD highly correlated with antenatal vitamin D (*r*=0.75) and was associated with ethnicity [Malay (*Β*=−4.44, *p*=2.2×10^−7^); Indian (*Β*=−1.99, *p*=0.038); reference Chinese]. GWAS analysis identified rs4588, a missense variant in the group-specific component (*GC*) gene encoding vitamin D binding protein (VDBP), and its defining haplotype, as a risk factor for low antenatal (*Β*=−8.56/T-allele, *p*=1.0×10^−9^) and cord blood vitamin D (*Β*=−3.22/T-allele, *p*=1.0×10^−8^) in all three ethnicities. We also discovered a novel association in a SNP downstream of *CYP2J2* (rs10789082), a gene involved in 25-hydroxylation of vitamin D, with vitamin D in pregnant women (*Β*=−7.68/G-allele, *p*=1.5×10^−8^), but not their offspring. As the prevention and early detection of suboptimal vitamin D levels are of profound importance to both mother and offspring’s health, the genetic risk variants identified in this study allow risk assessment and precision in early intervention of vitamin D deficiency.

## Introduction

Vitamin D is a steroid hormone that plays an important role in calcium homeostasis and metabolic pathways, and is hence linked with multiple human health outcomes (Theodoratou et al., 2014). The most abundant form of vitamin D is vitamin D3 (cholecalciferol), which is synthesized in the skin by exposure of 7-dehydrocholesterol to UV B radiation from sun. Alternatively, vitamin D, either as vitamin D2 (ergocalciferol) or vitamin D3, can also be consumed through diet. Vitamin D obtained from solar radiation is often influenced by spatiotemporal factors (latitude, altitude, seasonality, time of day, and air pollution) and individual-specific factors (skin pigmentation and preference for outdoor activity, clothing, and sunscreen use; Wacker and Holick, 2013).

After synthesis in the skin or consumption through diet, vitamin D circulates in the bloodstream and is rapidly converted to 25-hydroxycholecalciferol (25OHD) by cytochrome P450 (CYP) enzyme in the liver. Subsequent 1-hydroxylation in the kidney converts 25-hydroxycholecalciferol to the active metabolite 1,25-dihydroxycholecalciferol [1,25(OH)2D]. Vitamin D binding protein (VDBP) is the principal transporter of vitamin D and its metabolites in the blood stream and helps mobilize them to their target tissues. Many tissues express the vitamin D receptor (VDR), which binds to 1,25(OH)2D and heterodimerises with the retinoic X receptor (RXR) to form a transcription factor. Since RXR is involved in cell proliferation, differentiation, and organogenesis, it plays a critical role in pregnancy and fetal development (Szanto et al., 2004).

Measurement of circulating 25OHD, the major form of vitamin D in the bloodstream, is recommended to evaluate the vitamin D status (Holick et al., 2011). However, there is no consensus on cut-offs for deficient and insufficient vitamin D concentrations for pregnant women and newborns and even for the non-pregnant adult population. For example, the Institute of Medicine (IOM) guideline (Ross et al., 2011) defines vitamin D status as severely deficient (<30nmol/L), insufficient (30–49nmol/L), and sufficient (>50nmol/L), while the Endocrine Society Clinical Practice Guideline (Holick et al., 2011) defines vitamin D status as deficient (<50nmol/L), insufficient (50–75nmol/L), and sufficient (>75nmol/L). Various other thresholds are also used in practice (Nassar et al., 2011).

During pregnancy the maternal demand for calcium increases with calcification of the fetal skeleton, and this is corroborated with the increased circulation of maternal vitamin D and its metabolites. Furthermore, the fetus does not produce vitamin D and hence relies exclusively on the supply of maternal vitamin D through placenta. There is a strong correlation reported between infant cord blood and maternal 25(OH)D concentrations, especially toward late gestation and delivery (Sachan et al., 2005; Bodnar et al., 2007; Lee et al., 2007; Parlak et al., 2015; Rodda et al., 2015). The antenatal blood levels of VDBP have also been reported to increase by 40–50% during pregnancy, suggesting that VDBP may play a role in vitamin D homeostasis during gestation (Zhang et al., 2014; Karras et al., 2018). Thus, the heightened demand for vitamin D in pregnant women elevates their risk of developing vitamin D deficiency and many studies have shown high prevalence of vitamin D deficiency and insufficiency during pregnancy worldwide and in Asia (Sachan et al., 2005; Bodnar et al., 2007; Lee et al., 2007). The Endocrine Society Clinical Practice Guideline recommends that pregnant women require at least 600IU/day of vitamin D and may need 1,500–2,000IU/day to maintain their circulating 25OHD concentration of >75nmol/L (Holick et al., 2011).

Insufficient vitamin D concentrations during pregnancy have been associated with gestational diabetes, pre-eclampsia, bacterial vaginosis, antenatal and postnatal depression, and small for gestational age/low birth weight infants (Christesen et al., 2012; Aghajafari et al., 2013, 2018; Theodoratou et al., 2014). In this study involving the Growing Up in Singapore Towards healthy Outcomes (GUSTO) mother-offspring cohort (Soh et al., 2014), maternal vitamin D inadequacy was found to be associated with higher fasting glucose in Malays and increased risk of emergency cesarean section in Chinese and Indian women (Loy et al., 2015), as well as poor sleep quality and night-time eating during pregnancy (Cheng et al., 2017) and higher abdominal subcutaneous adipose tissue volume in the infants (Tint et al., 2018). However, although maternal vitamin D supplementation was shown to prevent neonatal vitamin D deficiency (Rodda et al., 2015), no evidence of benefit for pregnancy or birth outcomes have been demonstrated from vitamin D supplementation (Roth et al., 2015, 2017).

Genetic variation has been shown to contribute to vitamin D metabolism and risk of vitamin D insufficiency. Previous genome-wide association studies (GWAS) to identify risk factors for vitamin D have focused primarily on vitamin D measurements obtained from adults (Benjamin et al., 2007; Ahn et al., 2010; Wang et al., 2010; Moy et al., 2014; Manousaki et al., 2017; Jiang et al., 2018) and children (≥4years; Lasky-Su et al., 2012; Anderson et al., 2014) of European descent. Recently, two GWAS extended the investigations to include the African and Hispanic Americans populations (Hong et al., 2018; O’Brien et al., 2018). Only one GWAS has been conducted exclusively for vitamin D in an Asian population (Sapkota et al., 2016).

In this study, we measured vitamin D concentrations in the blood plasma of mothers of Chinese, Indian, and Malay descent in mid-pregnancy and in the cord blood of their offspring at birth. We investigate the epidemiological and genetic risk factors associated with antenatal and cord blood vitamin D concentrations. To our knowledge, this is the first GWAS investigating vitamin D concentrations of women during pregnancy, and their offspring at birth, and also in a multi-ethnic Asian cohort.

## Materials and Methods

### Study Population

Data were obtained from GUSTO, a mother-offspring prospective cohort study in Singapore (Soh et al., 2014). Briefly, 1,247 pregnant women with singleton pregnancies were recruited at 11–14weeks of gestation from two hospitals in Singapore, KK Women’s and Children’s Hospital (KKH) and National University Hospital (NUH), from June 2009 to September 2010. The inclusion criteria included age range between 18 and 50years, intention to reside in Singapore for the next 5years, intention to deliver in KKH and NUH, and willingness to donate antenatal and cord blood. We included only Chinese, Malay, and Indian women whose parents and whose partner’s parents were of the same ethnicity in the study. Informed written consent was obtained from all women. The study was conducted according to the guidelines laid down in the Declaration of Helsinki. Ethical approval was obtained from the Domain Specific Review Board of Singapore National Healthcare Group (reference D/09/021) and the Centralized Institutional Review Board of SingHealth (reference 2009/280/D).

### Maternal Characteristics

Demographic data on ethnicity, maternal age, educational levels, and pre-pregnancy weight was self-reported by participants at recruitment visit. Height, weight, and gestational diabetes status was measured at the GUSTO visit at 26–28weeks gestation. Briefly, women underwent a 75g oral glucose tolerance test (OGTT) and gestational diabetes was defined using the WHO 1999 definition (Alberti and Zimmet, 1998; fasting glucose ≥7.0mmol/L or 2-h post-OGTT glucose ≥7.8mmol/L). Mothers reported dietary supplements that they were consuming during pregnancy and trained research nutritionists contemporaneously coded the nutrient information from supplements to determine presence of vitamin D in supplements. The ethnicity of the study population was verified using the genotype data with the 1000 Genomes as reference population and samples not matching the self-reported ethnicity were removed.

### Infant Characteristics

Gestational age was assessed by ultrasonography in the first trimester in a standardized manner at both hospitals by trained ultrasonographers. Offspring sex and birthweight were obtained by trained research coordinators from birth records at the time of delivery.

### Antenatal Vitamin D Measurement

As described previously (Ong et al., 2016), maternal blood was collected during the clinical visit at 26–28weeks gestation (same time as OGTT) in EDTA tubes, centrifuged at 1,600*g* for 10min at 4°C within 4h of collection and the plasma was frozen at −80°C until analysis. Plasma 25OHD and its metabolite concentrations was analyzed by isotope-dilution liquid chromatography-tandem mass spectrometry (ID-LC-MS/MS). The intra- and inter-assay CVs for 25OHD were ≤10.3%, and the detection limit was <4nmol/L (Maunsell et al., 2005). The contribution of 25(OH)D2 was negligible (detected in <1% of antenatal samples). Thus, we used only 25(OH)D3 for analysis throughout this paper.

### Cord Blood Vitamin D Measurement

Cord blood was collected from infant umbilical cords either by directly dripping into EDTA tubes for normal deliveries, or extracted through a syringe for cords delivered through Cesarean section deliveries, then processed in the same way as maternal samples until analyses. Plasma 25OHD concentrations was analyzed in the laboratories of Bevital AS^1^ using LC-MS/MS (Midttun and Ueland, 2011). The intra- and inter-assay CVs for 25OHD were 4–5 and 7–8%, respectively, and the detection limit was 3.3nmol/L (Midttun and Ueland, 2011). The contribution of 25(OH)D2 was again negligible (detected in 1.3% of cord blood samples) and hence the subsequent analysis was restricted to 25(OH)D3 only.

### DNA Extraction, Genotyping, and Imputation

Mother’s DNA was extracted from blood collected at mid-gestation and infant DNA from cord tissue or blood and father’s DNA from buccal swabs. DNA extraction for mother and infants were as described previously (Lin et al., 2017). Isohelix DNA buccal swabs stored at −80°C were equilibrated to room temperature for 1h and 1ml of ATL lysis buffer (Qiagen) was added and incubated at room temperature for 30min. About 100μl of proteinase K (Qiagen) was then added and incubated at 60°C with shaking for 30min to 1h. DNA extraction from the lysates was performed using QIAsymphony DNA kits as per the manufacturer’s instructions.

The extracted DNA was genotyped using Illumina OmniExpress plus Exome array. DNA hybridization arrays and scanning were performed by Expression Analysis, Inc. (Morrisville, NC). Data were processed using GenomeStudio Genotyping Module version 1.0 (Illumina, Inc.). Briefly, genotyping calls were made by the GenCall software and genotypes with a GenCall score less than 0.15 are not assigned genotypes. Samples with genotyping call rate <97%, not matching self-reported ethnicity or discrepant in sex or with incongruent offspring-parent relationship (expected PI_HAT=0.5) were removed.

Genotype imputation was done for each ethnicity separately (for details and scripts^2^). Briefly, SNPs with minor allele frequency (MAF) <5%, call rate <95% or fail Hardy-Weinberg Equilibrium at value of *p*<10^−6^ (all parameters was estimated using parents only) were excluded in each ethnicity using PLINK version 1.90 (Chang et al., 2015). The data were aligned to GRCh37 build and further processed using a published pipeline^3^ before haplotype phasing using SHAPEIT2 with duoHMM method (O’Connell et al., 2014), which incorporates the family structure for better accuracy. We imputed the phased haplotypes with PBWT (Durbin, 2014) using the Sanger Imputation Service (McCarthy et al., 2016) using the 1000 Genomes Phase 3 (1000 Genomes Project Consortium et al., 2015) as reference panel. We analyzed 6,978,879 SNPs that passed stringent quality control (MAF>5% and imputation INFO>0.50) in at least one ethnicity.

### Statistical Analysis

25-hydroxyvitamin D was measured in 942 antenatal samples and 812 cord blood samples. While, we briefly report the proportion of women with sufficient concentrations (>75nmol/L) based on recommendations by Endocrine Society Clinical Practice Guideline (Holick et al., 2011), we analyze 25OHD concentrations as a continuous measure due to the lack of a well-established clinical cut-offs for insufficiency especially in pregnancy and for cord blood concentrations. Summary values are reported as mean±SD.

To identify significant covariates for the GWAS analysis, we first regressed the variable of interest with 25OHD using univariate linear regression models. Variables that were statistically significant (value of *p*<0.05) were included in a multivariate model and the most parsimonious model was selected using backward elimination procedure, i.e., start with the saturated model and drop a term if the *χ*^2^ value of *p*>0.05 for the reduction in Akaike’s information criteria.

Genome-wide association analysis for antenatal 25OHD was adjusted for ethnicity, consumption of vitamin D containing supplements and maternal age at recruitment. Similarly, GWAS for cord blood 25OHD was adjusting for antenatal 25OHD concentrations and ethnicity. SNPs were coded using the additive model of alleles. All analyses and plots were conducted in R version 3.3.2 unless stated otherwise. Associations reaching the genome-wide significance threshold (value of *p*≤5×10^−8^) were considered statistically significant and regional association plots using LocusZoom (Pruim et al., 2010) and ethnicity-stratified boxplots were generated. We also used Haploview (Barrett et al., 2005) to investigate the genetic linkage for selected loci. We attempted to elucidate the presence of second independent signals by including the selected SNP as a covariate in the model.

### Data Statement

Clinical data are not publicly available due to ethical restrictions but can be obtained from the authors upon reasonable request and subject to appropriate approvals from the GUSTO cohort’s Executive Committee.

## Results

### Vitamin D Inadequacy Is Very Common in Pregnant Mothers Especially in Malays and Indians

Vitamin D was measured at mid-gestation in 942 women (Supplementary Figure 1) from three major Asian ethnicities (520 Chinese, 175 Indian, and 247 Malay). 25(OH)D3 was the predominant vitamin D metabolite detected, with mean concentration of 81.08±27.16nmol/L (Supplementary Figure 2). Overall, 41% of the mothers had inadequate vitamin D (≤75nmol/L). In a multivariate regression model (Table 1), low concentrations of antenatal vitamin D strongly associated with being Malay (*Β*=−22.32nmol/L, *p*=2.3×10^−26^) or Indian (*Β*=−21.85, *p*=3.1×10^−21^) compared to Chinese mothers; not consuming supplements containing vitamin D (*Β*=−16.47, *p*=2.4×10^−13^) and being younger at recruitment (*Β*=0.47 per year, *p*=0.0058). These covariates collectively explained 21.1% of variability in antenatal vitamin D.

**Table 1:** Characteristics for antenatal vitamin D concentration.

	*N* (%)/mean (SD)	Univariate model, *p*	Multivariate model^b^	
			*Β*	*p*
*N*	942			
Antenatal 25OHD concentration in nmol/L, mean (SD)	81.08 (27.16)			
Ethnicity, *N* (%)				
Chinese	520 (55.2)	*ref*	*ref*	*ref*
Indian	175 (18.6)	1.1×10^−21^	−21.85	3.1×10^−21^
Malay	247 (26.2)	3.3×10^−28^	−22.32	2.3×10^−26^
Age in years at recruitment, mean (SD)	30.48 (5.05)	1.2×10^−7^	0.47	0.0058
Highest level of education attained, *N* (%)		0.076^c^		
Primary and secondary	285 (30.2)			
Post-secondary	324 (34.4)			
University	320 (34.0)			
Not answered	13 (1.4)			
Monthly income of household SGD, *N* (%)		0.0017^c^		*ns*
<$1,000	17 (1.8)			
$1,000–1,999	121 (12.8)			
$2,000–3,999	273 (29.0)			
$4,000–5,999	220 (23.4)			
≥$6,000	250 (26.5)			
Unknown or refused to answer	61 (6.5)			
Pre-pregnancy BMI in kg/m^2^, mean (SD)^a^	22.67 (4.29)	2.0×10^−5^		*ns*
Smoked or exposed to tobacco smoke during pregnancy, *N* (%)^d^	51 (5.4)	0.40		
Gestational weight gain in kg, mean (SD)^a^	8.62 (4.44)	0.59		
Gestational diabetes mellitus using WHO 1999, *N* (%)^a^	161 (17.7)	0.22		
Consumption of vitamin D containing supplements, *N* (%)				
Yes	700 (74.3)	*ref*	*ref*	*ref*
No	147 (15.6)	1.5×10^−7^	−16.47	2.4×10^−13^
Unknown	95 (10.1)			
Male offspring, *N* (%)	490 (52.1)	0.23		
With genotype data, *N* (%)	919 (97.6)			
With genotype data and information on supplements, *N* (%; i.e., sample size for Figure 1A and antenatal GWAS)	827 (87.8)			

a Contains missing values: pre-pregnancy BMI (*n*=78), gestational weight gain (*n*=80), AND gestational diabetes mellitus (*n*=34).

b For the multivariate model, we included all variables that was significant in an univariate test (*p*<0.05) followed backward elimination. Pre-pregnancy BMI and household income was not significant in the multivariate model and thus dropped in favor of a more parsimonious model (*ns,* not significant at *p*<0.05).

c Values of *p* shown is for the *F*-test from the ANOVA model.

d We considered a mother to be smoking during pregnancy if she self-reported to be smoking during pregnancy (*n*=29) or if the antenatal blood cotinine levels were >56.5nmol/L (*n*=20). We considered a mother to be exposed to tobacco smoke if her cotinine levels were between 11 and 56.5nmol/L (*n*=11).

**Figure 1:**
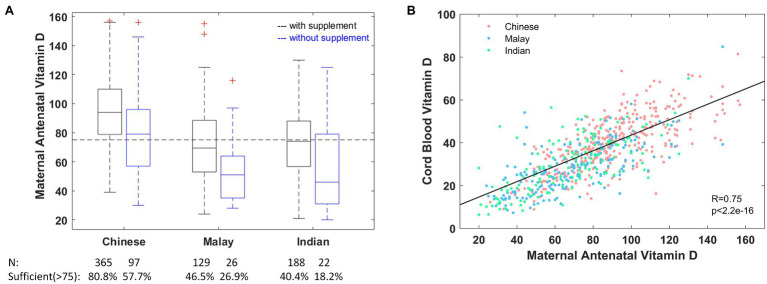
**(A)** Maternal antenatal vitamin D concentration stratified by ethnicity and consumption of vitamin D containing supplements. **(B)** Cord blood vitamin D concentration is strongly correlated with maternal antenatal vitamin D across all ethnicities. The dashed horizontal line marks the level of sufficiency (>75nmol/L) as recommended by the Endocrine Society Clinical Practice Guidelines (ESCPG; Holick et al., 2011). For visualization purposes, the *y* axis in **(A)** is truncated at 160nmol/L, which excludes two high values (187, 195) from Chinese mothers who consumed vitamin D containing supplements.

Majority of the mothers (84.6%) reported consuming supplements containing vitamin D and despite this 36.8% of these mothers (59.6% in Malay, 53.5% in Indian, and 19.2% in Chinese mothers; Figure 1A) had inadequate antenatal vitamin D. As expected, the proportion of pregnant women with vitamin D inadequacy is much higher at 53.8% among those not consuming vitamin D containing supplements (81.8% in Malay, 73.1% in Indian, and 42.3% in Chinese mothers).

### Cord Blood Vitamin D Is Highly Correlated With Maternal Antenatal Concentrations

Vitamin D was measured in cord blood from 812 infants (Supplementary Figure 1), and again the predominant vitamin D metabolite detected was 25(OH)D3, with a mean concentration of 34.05±13.76nmol/L (Supplementary Figure 2). In a multivariate regression model (Table 2), low concentration of cord blood vitamin D was strongly associated with being Malay (*Β*=−4.44, *p*=2.2×10^−7^) or Indian (*Β*=−1.99, *p*=0.038) compared to Chinese offspring. These covariates collectively explain 57.4% of variability in cord blood vitamin D. There was a high correlation between cord blood and maternal antenatal vitamin D with a Pearson correlation of 0.75 (Figure 1B) and this correlation persisted even when stratified by ethnicity (0.70 in Chinese, 0.69 in Indian, and 0.70 in Malay).

**Table 2:** Characteristics for cord blood vitamin D levels.

	*N* (%)/mean (SD)	Univariate model, *p*	Multivariate model^b^	
			Β	*p*
*N*	812			
Cord blood 25OHD levels in nmol/L, mean (SD)	34.05 (13.76)			
Antenatal 25OHD levels in nmol/L, mean (SD)^a^	79.00 (27.29)	2.0×10^−120^	0.35	9.6×10^−99^
Ethnicity, *N* (%)				
Chinese	399 (49.1)	*ref*	*ref*	*ref*
Indian	166 (20.4)	4.0×10^−15^	−1.99	0.038
Malay	247 (30.4)	8.3×10^−29^	−4.44	2.2×10^−7^
Gestational age in weeks, mean (SD)	38.75 (1.36)	0.69		
Birth Weight in kg, mean (SD)	3.10 (0.44)	0.073		
Male offspring, *N* (%)	432 (53.3)	0.88		
With genotype data, *N* (%)	777 (95.6)			
With genotype data and mother’s antenatal 25OHD measurement, *N* (%; i.e., sample size for Figure 1B and cord blood GWAS)	656 (80.8)			

a Contains missing values: Antenatal vitamin D (*n*=144).

b For the multivariate model, we included all variables that was significant in an univariate test (*p*<0.05) followed backward elimination.

### A Missense Variant (rs4588) in *GC* Is Associated With Decreased Concentrations of Vitamin D

**Figure 2:**
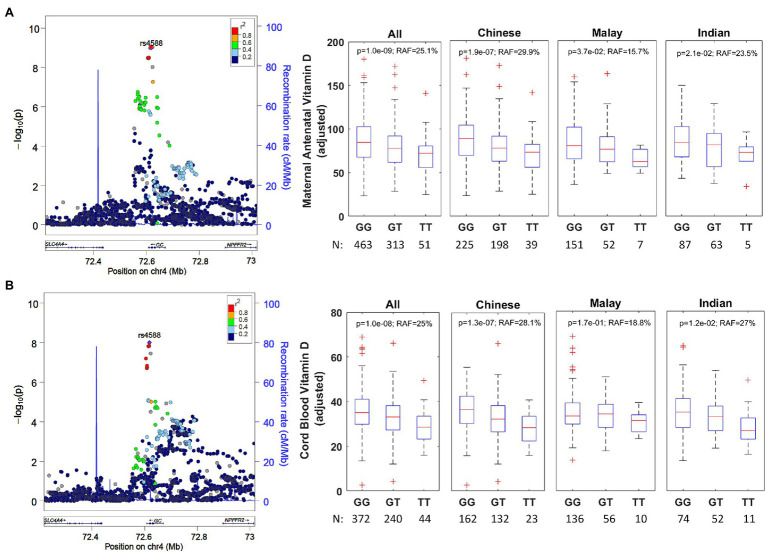
**(A)** Regional association plot for rs4588 group-specific component (*GC*) and ethnicity-stratified levels for antenatal vitamin D. The regional association plot (left) is based on all individuals with complete data for analysis. The data presented as boxplots (right) has been adjusted for ethnicity, mother’s age at recruitment and consumption of vitamin D containing supplements. RAF, risk allele frequency. **(B)** Regional association plot for rs4588 (*GC*) and ethnicity-stratified levels for cord blood vitamin D. The regional association plot (left) is based on all individuals with complete data for analysis. The data presented as boxplots (right) has been adjusted for ethnicity and antenatal vitamin D levels.

We conducted GWAS for vitamin D concentrations measured in 827 pregnant women and 656 cord blood samples (Supplementary Figures 1–4 for sample selection flow diagram, phenotype distribution, QQ, and Manhattan plots). The strongest signal for maternal antenatal and cord blood vitamin D (Table 3) was in the *GC* gene, which encodes the VDBP (Figure 2).

**Table 3:** Variants reaching genome-wide significance.

Gene	dbSNP	Chr	Position	Reference allele	Risk allele	Risk allele frequency	*Β*	*p*	Source
**Variants reaching genome-wide significant with antenatal vitamin D concentration:**									
*CYP2J2*	rs10789082	1	60,357,969	A	G	29.4%	−7.68	1.5×10^−8^	Typed
*GC*	rs17467825	4	72,605,517	A	G	24.9%	−8.53	3.3×10^−9^	Imputed
*GC*	rs2282680	4	72,608,364	C	T	24.7%	−8.41	3.4×10^−9^	Imputed
*GC*	rs2282679	4	72,608,383	T	G	24.7%	−8.36	3.2×10^−9^	Typed
*GC*	rs3755967	4	72,609,398	C	T	24.7%	−8.37	3.1×10^−9^	Imputed
*GC*	rs2298850	4	72,614,267	G	C	25.1%	−8.58	9.8×10^−10^	Imputed
*GC*	rs11723621	4	72,615,362	A	G	25.1%	−8.58	9.9×10^−10^	Imputed
*GC*	rs1352846	4	72,617,775	A	G	24.1%	−8.88	6.5×10^−10^	Imputed
*GC*	rs4588	4	72,618,323	G	T	25.1%	−8.56	1.0×10^−9^	Typed
**Variants reaching genome-wide significant with cord blood vitamin D concentration:**									
*GC*	rs2298850	4	72,614,267	G	C	25.0%	−3.18	1.5×10^−8^	Imputed
*GC*	rs11723621	4	72,615,362	A	G	25.0%	−3.18	1.5×10^−8^	Imputed
*GC*	rs1352846	4	72,617,775	A	G	24.6%	−3.22	1.5×10^−8^	Imputed
*GC*	rs4588	4	72,618,323	G	T	25.0%	−3.22	1.0×10^−8^	Typed

The antenatal vitamin D GWAS identified an intronic variant rs1352846 in *GC* as the most statistically significant association (*p*=6.5×10^−10^). However, this variant is in a very high linkage disequilibrium (*r^2^*=0.957 in Chinese, *r^2^*=0.983 in Indian, and *r^2^*=0.985 in Malay; Supplementary Figure 5) with rs4588 (*p*=1.0×10^−9^), which is a missense variant that has been previously reported to be associated for vitamin D in European, African American, and Hispanic descent populations (Lasky-Su et al., 2012; Anderson et al., 2014; Hong et al., 2018; Jiang et al., 2018; O’Brien et al., 2018). rs4588 was also the most significant risk variant associated with cord blood vitamin D concentrations (*p*=1.0×10^−8^). Therefore, we postulate rs4588 is most likely the causal variant in GC. There was no secondary signal in *GC* gene after conditioning on rs4588. The T-allele is the risk allele for rs4588 and is associated with a reduction of 8.6nmol/L per allele in antenatal vitamin D (*p*=1.0×10^−9^) and a reduction of 3.2nmol/L per allele in cord blood vitamin D (*p*=1.0×10^−8^). This variant explains 3.3 and 2.0% of the variability in antenatal and cord blood vitamin D, respectively, after accounting for the factors included in multivariate models in Tables 1 and 2. The directionality of this association is consistent across ethnicities (Figure 2), while the risk allele frequency (RAF) varies from 29.9% in Chinese, 23.5% in Indian, and 15.7% in Malay mothers.

The missense variant rs4588 (p.Thr436Lys) and missense variant rs7041 (p.Glu432Asp) are frequently studied together as haplotype-tagging SNPs in *GC*. While rs7041 only had a modest statistical association in our single-SNP GWAS (*p*=2.8×10^−4^ for antenatal and *p*=0.01 for cord blood), the results for haplotype association (Table 4) were highly significant (ANOVA *p*=2.0×10^−7^ for antenatal and *p*=3.0×10^−6^ for cord blood). We find that the vitamin D concentrations are highest in individuals with haplotypes 1s/1s or 1s/1f and lowest in those with haplotype 2/2, in agreement with the findings of a large study with 11,704 Norwegian adults (Jorde and Grimnes, 2015).

**Table 4:** Analysis of *GC* haplotypes for the antenatal and cord blood vitamin D genome-wide association analysis (GWAS).

Haplotype and definition			Antenatal vitamin D (*N*=827)^a^				Cord blood vitamin D (*N*=656)^b^				Published values for Jorde and Grimnes (2015)	
Haplotype	rs4588^c^	rs7041^c^	*N*	mean±SD	*Β*	*p*	*N*	mean±SD	*Β*	*p*	*N*	mean±SD
1s/1s	GG	CC	114	86.7±24.8	*ref*	*ref*	99	35.5±9.2	*ref*	*ref*	3,621	55.4±16.8
1s/1f	GG	AC	210	86.5±24.8	−1	0.74	147	35.9±8.4	0.43	0.72	2,456	53.3±17.2
1f/1f	GG	AA	139	85.6±24.9	−1.78	0.59	126	35.7±9.1	0.23	0.85	510	52.2±16.8
1s/2	GT	AC	150	80.1±24.8	−7.45	0.015	113	33.2±8.7	−2.36	0.052	3,315	50.3±15.6
1f/2	GT	AA	163	76.5±20.4	−11.3	3.4×10^−4^	127	32.6±8.6	−2.97	0.016	1,104	50.5±16.3
2/2	TT	AA	51	69.7±20.9	−18.3	1.6×10^−5^	44	28.9±7.1	−6.67	3.7×10^−5^	698	46.9±15.1

a Mutually adjusted for ethnicity, consumption of vitamin D containing supplements and age at recruitment. The mean±SD column is after adjusting for these covariates. The B and value of *p* are from the estimates from the linear regression.

b Mutually adjusted for antenatal vitamin D levels and ethnicity. The mean±SD column is after adjusting for these covariates. The *B* and value of *p* are from the estimates from the linear regression.

c All of the alleles are reported on the positive strand.

### A Variant (rs10789082) Downstream of *CYP2J2* Is Associated With Decreased Antenatal Vitamin D

**Figure 3:**
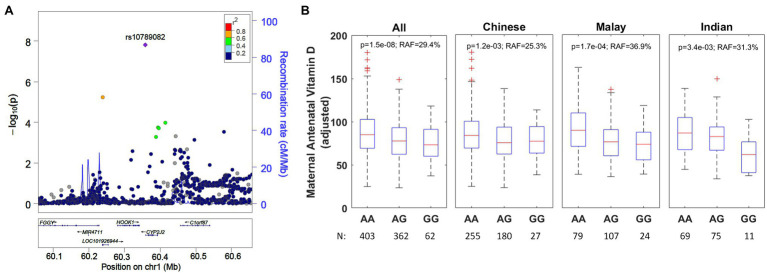
**(A)** Regional association plot for rs10789082 (*CYP2J2*) and ethnicity-stratified levels for antenatal vitamin D. The regional association plot is based on all individuals with complete data for analysis. **(B)** Boxplots adjusted for ethnicity, mother’s age at recruitment, and consumption of vitamin D containing supplements. RAF, risk allele frequency.

The second strongest genetic association was for the SNP rs10789082 (Table 3; Figure 3), which is located 1,011 bases downstream from the 3' end of the last exon (exon 9, Supplementary Figure 6) of the *CYP2J2* (*Cytochrome P450 Family 2 Subfamily J Member 2*) gene. The G-allele is the risk allele for rs10789082 and is associated with a reduction of 7.7nmol/L per allele in antenatal vitamin D (*p*=1.5×10^−8^) but this SNP was not associated with cord blood vitamin D (*p*=0.65). This variant explains an additional 2.0% of the variability in antenatal vitamin D after accounting for the factors included in multivariate models in Table 1. We note that this SNP is not in high linkage disequilibrium with any of the SNPs in the region of the *CYP2J2* gene but this variant is a genotyped SNP with good separation of the allele signals for genotype calling (Supplementary Figure 7). Furthermore, the directionality of this association is consistent across ethnicities (Figure 3).

## Discussion

In this mother-offspring study of three major Asian ethnicities (Chinese, Indian, and Malay), which represents 40% of the global population, we measured the vitamin D concentrations in blood collected mid-pregnancy and from the umbilical cord of their offspring. First, we found that many pregnant women, particularly those of Malay and Indian descent, do not achieve the recommended concentrations of 75nmol/L and above of vitamin D in pregnancy. We also observed a substantial level of vitamin D insufficiency despite majority of mothers reporting consumption of vitamin D containing supplements. This discrepancy may be due to the low amounts of vitamin D in the supplements consumed as there is a heightened demand for vitamin D during pregnancy and up to 1,500–2,000IU/day may be needed to maintain their circulating 25OHD concentration of >75nmol/L (Holick et al., 2011). An additional reason could be the limitations of the self-reporting questionnaire to collect details of duration of supplement consumption and compliance to the recommended dosage. Next, we found that the vitamin D concentrations in cord blood are strongly correlated with the mothers’ mid-pregnancy vitamin D concentrations across all three ethnicities. Mothers with inadequate vitamin D concentrations and their offspring are at risk of pregnancy complications and early life adverse outcomes (Nassar et al., 2011; Christesen et al., 2012; Aghajafari et al., 2013, 2018; Theodoratou et al., 2014). To develop more insights into vitamin D insufficiency, we examined the genetic association with antenatal and cord blood vitamin D concentrations, which revealed two interesting loci.

Our GWAS identified rs4588, a missense variant in the gene *GC* of both mother and the offspring, as a significant risk factor for decreased vitamin D concentrations in antenatal and cord blood. The key haplotype-tagging SNP rs4588 has been previously reported as a risk factor for vitamin D in adult and non-pregnant populations of European, African American, and Hispanic descent (Manousaki et al., 2017; O’Brien et al., 2018). Several other variants in *GC* (rs2282679, rs3755967, and rs17467825) in high linkage disequilibrium with rs4588 have also been associated with vitamin D concentrations previously (Ahn et al., 2010; Wang et al., 2010; Lasky-Su et al., 2012; Anderson et al., 2014; Hong et al., 2018; Jiang et al., 2018). The *GC* gene encodes the VDBP, a protein that stores and transports both 25OHD and the active form of vitamin D, 1,25(OH)2D (Speeckaert et al., 2006), thus variants affecting VDBP activity would likely influence measured circulating 25OHD concentrations. Our findings augment the results of previous GWAS on vitamin D. The rs4588 and rs7041 haplotype GC 1F has been shown to affect VDBP binding to vitamin D (Arnaud and Constans, 1993), thus may affect the half-life of 25OHD and/or the bioavailability of free 25OHD. The majority of circulating 25OHD is bound to VDBP (Karras et al., 2018). Pregnant women have increased VDBP due to estrogen-dependent production and have increased 25OHD concentrations likely due to higher concentrations of VDBP and/or vitamin D supplementation (Karras et al., 2018). The effects of dysregulation of VDBP may play a role in the associations of vitamin D insufficiency and adverse pregnancy-related outcomes (Karras et al., 2018).

We also identified rs10789082 downstream of *CYP2J2* to be associated with antenatal vitamin D. This is a novel association in humans and is consistent across all three ethnicities. It is interesting to find this association with antenatal vitamin D concentrations but not cord blood vitamin D concentrations. This may be because the fetus depends on the mother for 25-hydroxylation of vitamin D. 25OHD crosses the placenta and fetal vitamin D concentrations are dependent on maternal vitamin D concentrations (Salle et al., 2000; Hollis and Wagner, 2017). The fetus appears to obtain active 1,25OHD largely from fetal kidney activity but 1,25OHD may also cross the placenta (Salle et al., 2000). SNPs in *CYP2J2* have been reported to be associated with serum vitamin D status in beef cattle (Casas et al., 2013) but not in human. The gene *CYP2J2* encodes the enzyme CYP, family 2, subfamily J, and polypeptide 2. The CYP superfamily of enzymes catalyze the oxidation of small organic compounds and are involved in drug metabolism and activation of steroid hormones such as vitamin D. The human CYP2J2 enzyme has been demonstrated to hydroxylate vitamin D2, vitamin D3, and 1α-hydroxyvitamin D3 (Aiba et al., 2006). SNPs in related genes *CYP2R1* and *CYP24A1* have been previously associated with vitamin D concentrations (Ahn et al., 2010; Wang et al., 2010; Hong et al., 2018; Jiang et al., 2018; O’Brien et al., 2018) but these reported associations were not significant in our cohort (*p*>0.01). 25-hydroxylation of vitamin D is thought to primarily be due to CYP2R1 with CYP27A1, CYP2J2, and CYP3A4 also contributing and subsequent 1-alpha-hydroxylation by CYP27B140. Perhaps CYP2J2 plays a larger role in 25-hydroxylation of vitamin D during pregnancy or in Asian populations.

The present study has several strengths. To our knowledge, this is the first GWAS to investigate vitamin D in the context of antenatal blood, paired offspring cord blood and also in a multi-ethnic Asian cohort. Our cohort offers the unique opportunity to compare three major Asian ethnicities in a standardized manner as all participants live in a geographically small region with constant sunshine, little seasonal variation, and, where data was collected in a uniform manner. We used the LC-MS/MS method to quantify vitamin D concentrations, which is the proposed reference method for 25OHD measurement (Vogeser et al., 2004), as the competitive binding assays commonly used for measuring 25OHD may underestimate vitamin D concentrations due to differences in antibody affinity (Ong et al., 2012).

There are also some limitations to this study. One limitation is the single measurement of vitamin D in pregnancy, as it is known that pregnancy is marked by dynamic changes in physiology and vitamin D increases during the course of pregnancy. However, the time point chosen for this study is mid-gestation, which corroborates with the rise in antenatal vitamin D levels and fetal bone development (Moon et al., 2015). We have not measured the physiologically active form of vitamin D, 1,25(OH)2D, which has been shown to increase by 100% or more during pregnancy (Christesen et al., 2012); however, 25(OH)D reflects vitamin D stores and is the form of vitamin D recommended to evaluate vitamin D status (Holick et al., 2011). Second, we have not accounted for diet in our model, although, we anticipate sun exposure to have a greater role in determining vitamin D concentrations as the major vitamin D metabolite detected in plasma was 25(OH)D3. Third, we have also not accounted for individual-specific factors (skin pigmentation, preference for outdoor activity, and clothing preferences) in our models. We observe that the ethnicity specific effects are still present after accounting for the genetic associations identified here, suggesting that these factors may explain some of the difference in vitamin D concentrations between ethnic groups. Fourth, although, we adjusted for reported consumption of vitamin D containing supplements, we are unable to adjust for exact amounts consumed as we cannot ascertain the compliance and frequency of consuming the supplements with high degree of confidence. Fifth, we did not have weather data on amount of sunshine, although Singapore is a small island lying 1.5° north of the equator with sunshine throughout the year with no true distinct seasons. Therefore, we should anticipate very little impact due to the usual spatiotemporal factors that might affect the participants. Finally, the genotype association with rs10789082 downstream of *CYP2J2* needs to be replicated in other studies, preferably in Asian ethnicities first.

In conclusion, we found high prevalence of vitamin D inadequacy in pregnant women in a country with predominantly Asian population receiving sunlight all year round. Both Malay and Indian ethnic groups had more vitamin D inadequacy compared to Chinese. Our genetic finding on VDBP, the primary transporter of vitamin D in circulation, augments the understanding of vitamin D deficiency in pregnancy and its downstream impact on the availability of vitamin D to the growing fetus when the demands for it are critical during gestation. We have also identified a new SNP downstream of *CYP2J2*, which may influence vitamin D concentrations in pregnancy. The association of these genetic risk factors are similar across all three Asian ethnic groups studied here. These results may help to identify individuals who are genetically predisposed to vitamin D insufficiency in Asian population and advise further studies of the impact of this genetic predisposition on the association between vitamin D and different pregnancy and child health outcomes.

## Data Availability Statement

Clinical data are not publicly available due to ethical restrictions but can be obtained from the authors upon reasonable request and subject to appropriate approvals from the GUSTO cohort’s Executive Committee.

## Ethics Statement

The study was conducted according to the guidelines laid down in the Declaration of Helsinki. Ethical approval was obtained from the Domain Specific Review Board of Singapore National Healthcare Group (reference D/09/021) and the Centralized Institutional Review Board of SingHealth (reference 2009/280/D). Written informed consent to participate in this study was provided by the participants’ legal guardian/next of kin.

## Author Contributions

AS, AR, and LC performed the statistical analysis and genome-wide associations. AR, KT, and NK interpreted the data and wrote the manuscript. YC, PG, FY, KG, MC, and NK were responsible for data generation (clinical and genotyping data) in GUSTO cohort. NK conceptualized and supervised the study. All authors contributed to the article and approved the submitted version.

## Funding

This research is supported by the Singapore National Research Foundation under its Translational and Clinical Research (TCR) Flagship Program on Developmental Pathways to Metabolic Disease and administered by the Singapore Ministry of Health’s National Medical Research Council (NMRC), Singapore-NMRC/TCR/004-NUS/2008; NMRC/TCR/012-NUHS/2014. KMG is supported by the UK Medical Research Council (MC_UU_12011/4), the National Institute for Health Research (NIHR Senior Investigator (NF-SI-0515-10042) and NIHR Southampton Biomedical Research Centre (IS-BRC-1215-20004)), the European Union (Erasmus+ Programme ImpENSA 598488-EPP-1-2018-1-DE-EPPKA2-CBHE-JP) and the British Heart Foundation (RG/15/17/3174, SP/F/21/150013). Additional funding is provided by the Singapore Institute for Clinical Sciences (SICS), Joint Council Office (JCO) Grant (JCO1431AFG110), and Strategic Positioning Fund (SPF) awarded to NK by the Agency for Science, Technology and Research (A*STAR), Singapore.

## Conflict of Interest

YC and KG have received reimbursement for speaking at conferences sponsored by companies selling nutritional products. YC, KG, and NK are part of an academic consortium that has received research funding from Abbott Nutrition, Nestec, Evolve Biosystems and Danone.

The remaining authors declare that the research was conducted in the absence of any commercial or financial relationships that could be construed as a potential conflict of interest.

## Publisher’s Note

All claims expressed in this article are solely those of the authors and do not necessarily represent those of their affiliated organizations, or those of the publisher, the editors and the reviewers. Any product that may be evaluated in this article, or claim that may be made by its manufacturer, is not guaranteed or endorsed by the publisher.

## Footnotes

^1^ www.bevital.no

^2^ https://bitbucket.org/sics-sb/gusto-imputation.git

^3^ https://github.com/natacha-beck/imputePrepSanger
