# Supplementary material for: Genetic Link Determining the Maternal-Fetal Circulation of Vitamin D
Source: Front Genet. 2021 Sep 21;12:721488. doi: 10.3389/fgene.2021.721488 (PMC8490770; doi:10.3389/fgene.2021.721488)
Supplement: Supplementary file 1 [file Data_Sheet_1.docx]

Supplementary Material

## Supplementary Figures


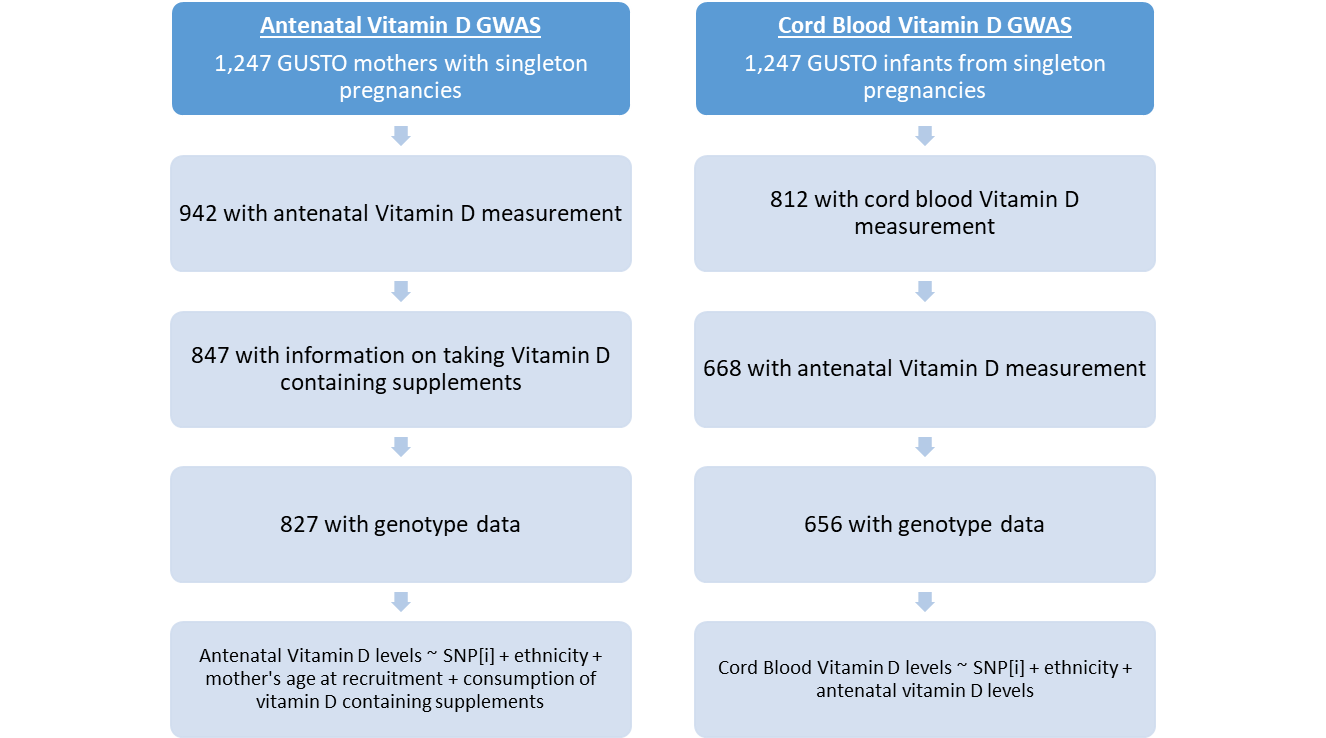


**Supplementary Figure 1.** Flow chart to show how the final sample size used for GWAS was derived.


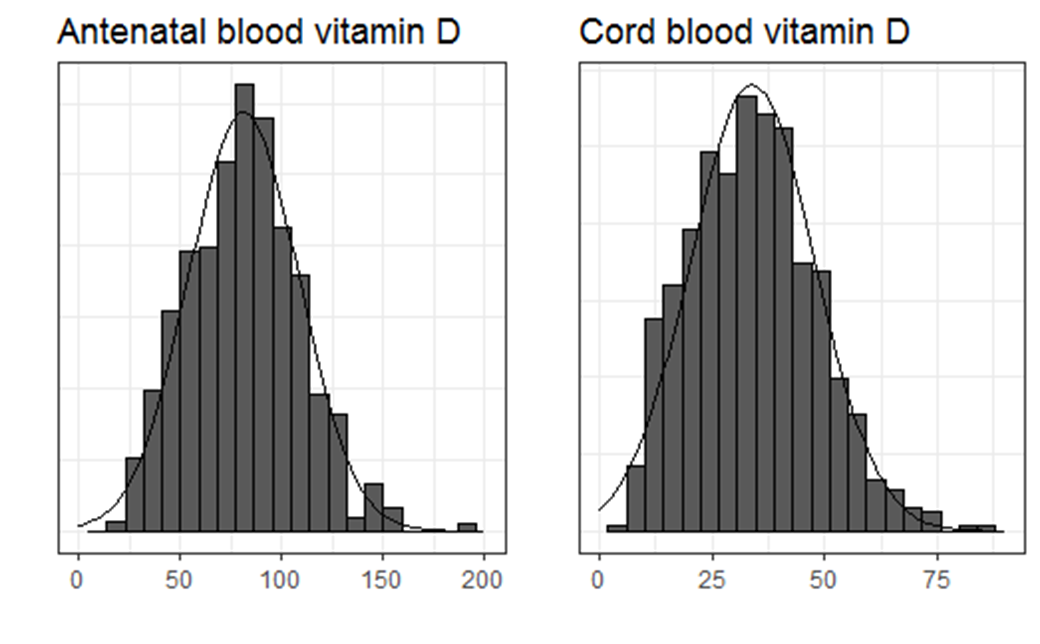


**Supplementary Figure 2.** Distribution of the measured values for antenatal and cord blood vitamin D in nmol/L.

**Antenatal Vitamin D (trans-ethnic) Cord Blood Vitamin D (trans-ethnic)**
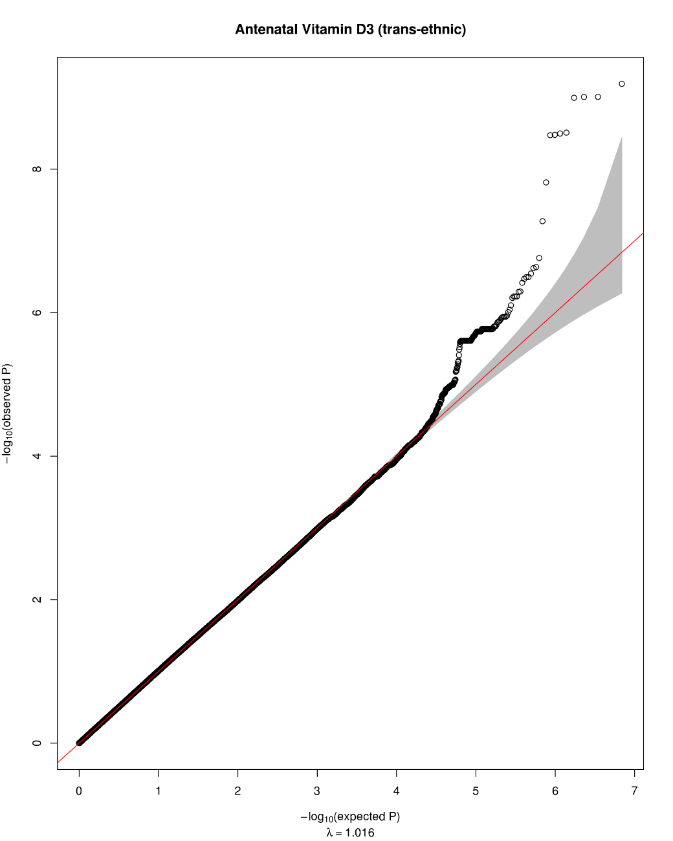

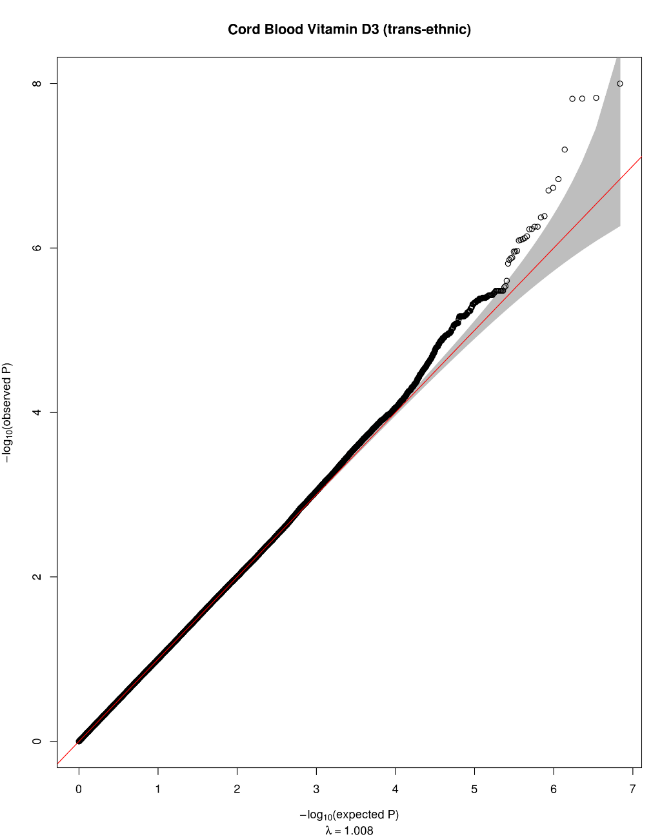


**Supplementary Figure 3.** QQ plot for the antenatal (left) and cord blood (right) GWAS on vitamin D.


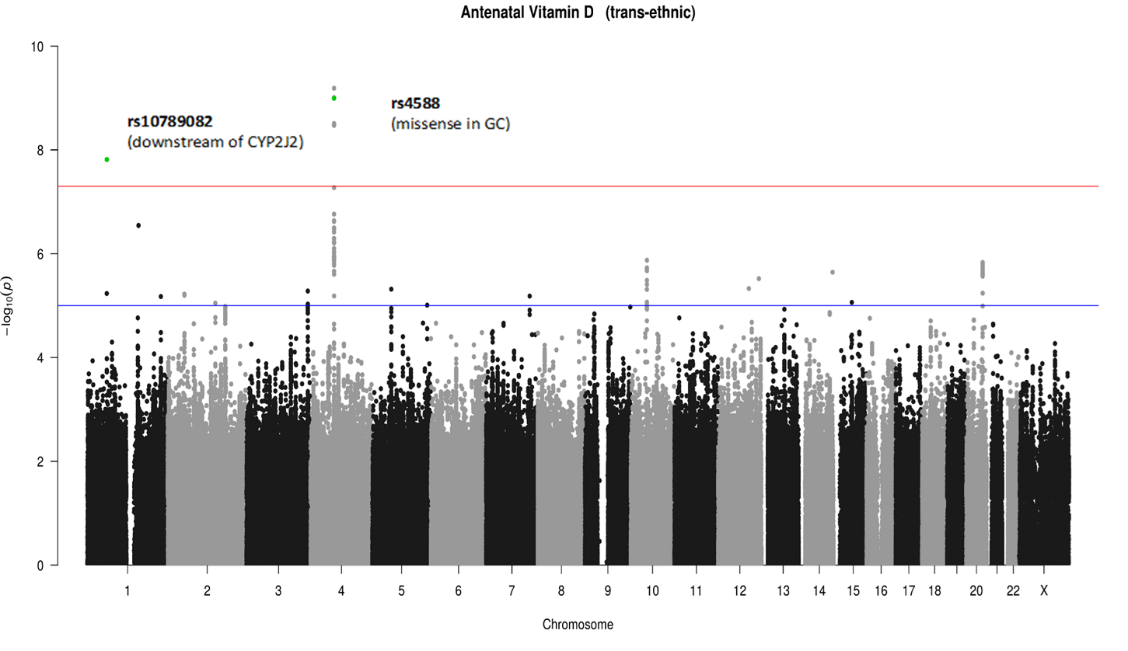


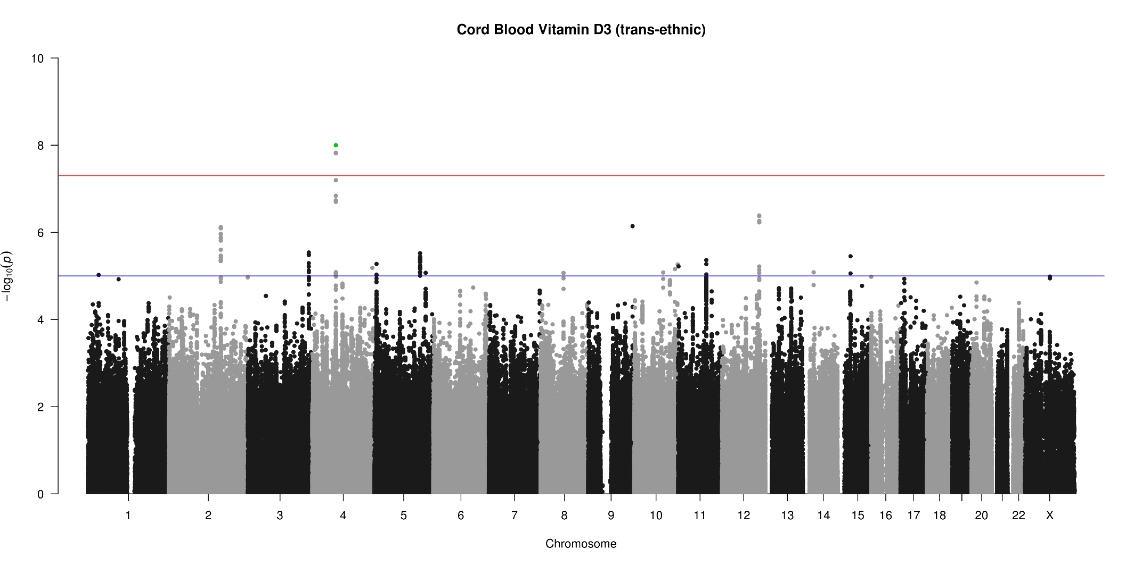


**rs4588**

(missense in *GC*)

**Supplementary Figure 4.** Manhattan plots for GWAS for vitamin D levels in antenatal and cord blood in trans-ethnic model. The red line in the Manhattan plot depicts the genome-wide significance threshold of 5 x 10^-8^ and the blue line depicts the suggestive threshold of 1 x 10^-5^.


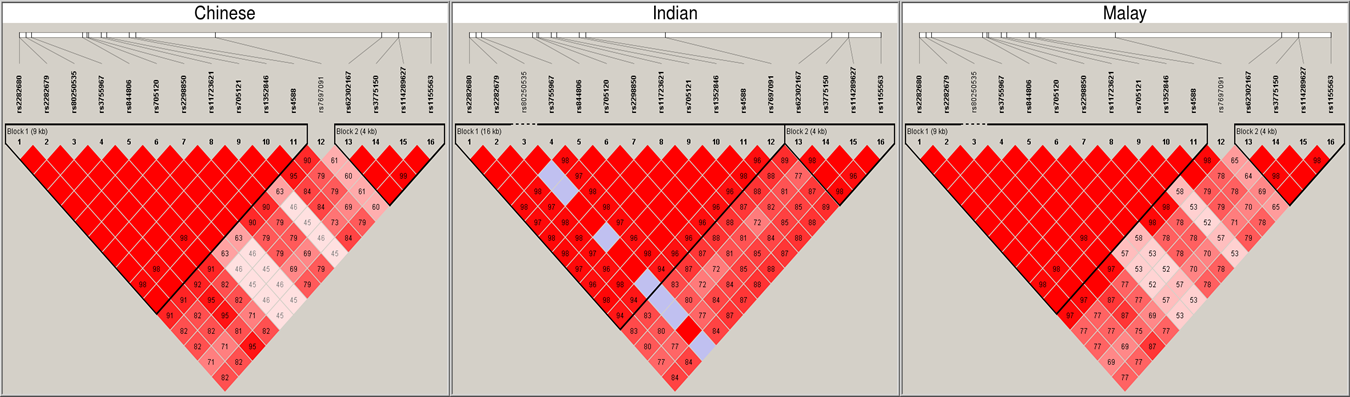


**Supplementary Figure 5.** The linkage disequilibrium plot using R^2^ with the 16 SNPs with *p* < 10^-5^ in the antenatal vitamin D (12 of which also achieve *p* < 10^-5^ in cord blood vitamin D) in the *GC* gene **region.** Similar haplotype blocks are seen across the three ethnicities among the selected SNPs. All of the variants that pass genome-wide significance in **antenatal** vitamin D (see Table 3) are located in Block 1: rs1352846 (*p*=6·5x10^-10^), rs2298850 (*p*=9·8 x10^-10^), rs11723621 (*p*=9·90 x10^-10^), **rs4588** (*p*=1·00 x10^-9^), rs3755967 (*p*=3·1x10^-9^), rs2282679 (*p*=3·2x10^-9^), rs17467825 (*p*=3·3x10^-9^), rs2282680 (*p*=3·4x10^-9^). All of the variants that pass genome-wide significance in **cord blood** vitamin D (see Table 3) are located in Block 1: **rs4588** (*p*=1·0x10^-8^), rs2298850 (*p*=1·5x10^-8^), rs11723621 (*p*=1·5x10^-8^), rs1352846 (*p*=1·5x10^-8^).


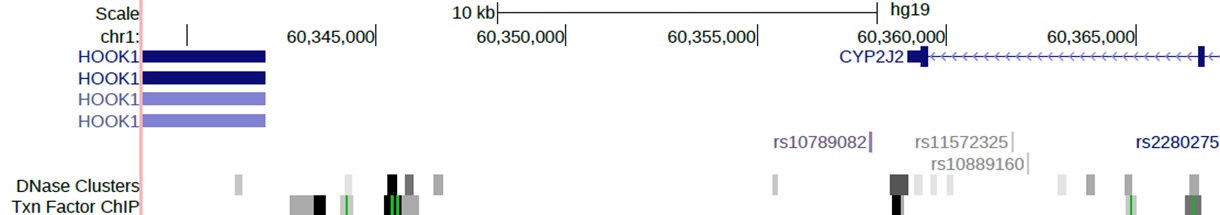


**Supplementary Figure 6.** Snapshot of UCSC genome browser depicting the SNP rs10789082 (boxed in red) in relation to the *CYP2J2* gene.


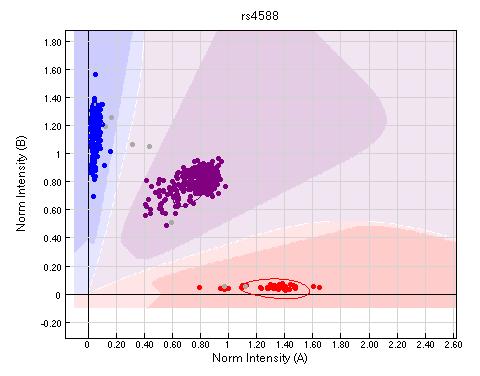

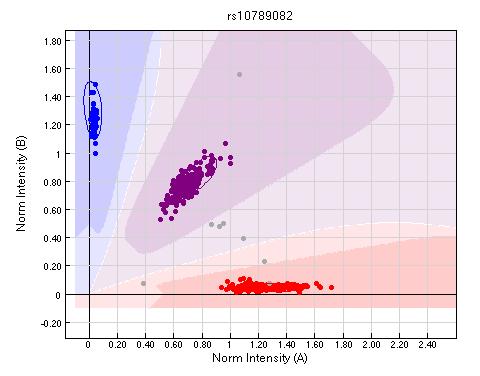


**Supplementary Figure 7.** The allele intensity signals for the two significant SNPs identified in GUSTO mothers that are typed on the genotyping chip showing good separation of the signals indicating accurate genotype calling.
